# Methylation-regulated ZNF545 inhibits growth of the p53-mutant KYSE150 cell line by inducing p21 and Bax

**DOI:** 10.3892/etm.2021.9948

**Published:** 2021-03-22

**Authors:** Yu Fan, Yu Wang, Shaozhi Fu, Duan Liu, Sheng Lin

Exp Ther Med 18:1563-1570, 2019; DOI: 10.3892/etm.2019.7737

Subsequently to the publication of this article, the authors have realized that they were unable to replicate the data shown in Fig. 1C in repeated experiments; moreover, the wrong MSP images had been incorporated into Fig. 1C, and the correct data were lost.

All authors agreed that the problem with these data could be rectified by simply omitting the affected Figure part, and that this would not have a major impact on the conclusions reported in the paper. The corrected version of Fig. 1, with the omission of Fig. 1C and its revised legend, are shown opposite. As a consequence of the correction to the figure, note that the pair of sentences in the Results section on p. 1565, right-hand column, starting from line 9 should now read as follows: “The promoter methylation status of ZNF545 in tumor tissues (n=64) and adjacent normal tissues (n=64) was also evaluated using an MSP assay. ZNF545 promoter methylation was observed in 76.6% (49/64) of primary tumors, compared with only 28.1% (28/64) of adjacent normal tissues (P<0.001; Table II).” All the authors agree with the publication of this Corrigendum, regret this error that was only noticed subsequently to the publication of the article, and apologize to the readership for any inconvenience caused.

## Figures and Tables

**Figure 1:**
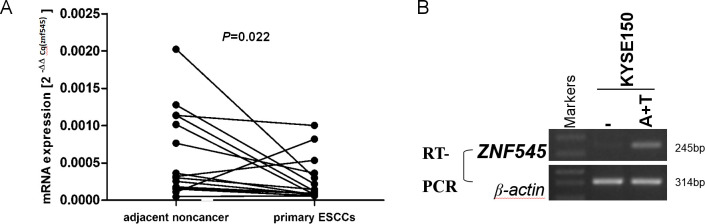
ZNF545 expression is downregulated by methylation in ESCC tissues. (A) ZNF545 mRNA expression was significantly reduced in primary ESCC tissues when compared with adjacent noncancer samples (n=15), according to reverse transcription-quantitative PCR analysis. (B) ZNF545 mRNA expression was restored in KYSE150 cells following treatment with the demethylating reagent, 5-Aza-2’-deoxycytidine, and the histone deacetylase inhibitor, trichostatin A. ZNF545, zinc finger protein 545; ESCC, esophageal squamous cell carcinoma; A + T, 5-Aza-2’-deoxycytidine and trichostatin A.

